# Long Noncoding RNAs, Chromatin, and Development

**DOI:** 10.1100/tsw.2010.7

**Published:** 2010-01-08

**Authors:** Daniel P. Caley, Ryan C. Pink, Daniel Trujillano, David R. F. Carter

**Affiliations:** ^1^ School of Life Sciences, Oxford Brookes University, UK; ^2^ Cranfield Health, Cranfield University, UK; ^3^ Genetic Causes of Disease Group, Genes and Disease Program, Center for Genomic Regulation (CRG-UPF), Barcelona, Spain

**Keywords:** long noncoding RNAs, lncRNAs, lincRNAs, noncoding RNAs, ncRNAs, chromatin, development, transcription, epigenetics, intergenic transcription, epigenomics

## Abstract

The way in which the genome of a multicellular organism can orchestrate the differentiation of trillions of cells and many organs, all from a single fertilized egg, is the subject of intense study. Different cell types can be defined by the networks of genes they express. This differential expression is regulated at the epigenetic level by chromatin modifications, such as DNA and histone methylation, which interact with structural and enzymatic proteins, resulting in the activation or silencing of any given gene. While detailed mechanisms are emerging on the role of different chromatin modifications and how these functions are effected at the molecular level, it is still unclear how their deposition across the epigenomic landscape is regulated in different cells. A raft of recent evidence is accumulating that implicates long noncoding RNAs (lncRNAs) in these processes. Most genomes studied to date undergo widespread transcription, the majority of which is not translated into proteins. In this review, we will describe recent work suggesting that lncRNAs are more than transcriptional "noise", but instead play a functional role by acting as tethers and guides to bind proteins responsible for modifying chromatin and mediating their deposition at specific genomic locations. We suggest that lncRNAs are at the heart of developmental regulation, determining the epigenetic status and transcriptional network in any given cell type, and that they provide a means to integrate external differentiation cues with dynamic nuclear responses through the regulation of a metastable epigenome. Better characterization of the lncRNA-protein "interactome" may eventually lead to a new molecular toolkit, allowing researchers and clinicians to modulate the genome at the epigenetic level to treat conditions such as cancer.

## INTRODUCTION

As the complexity of multicellular eukaryotes increases, it is reasonable to assume that so would the number of genes that orchestrate their development. The results of many genome-sequencing projects, however, are challenging this expectation. Humans contain 500 billion times more cells than the humble nematode worm, *Caenorhabditis elegans*, as well as a significantly more complex distribution of cell types, yet both organisms contain approximately the same number of genes, ~20,000[1,2]. The use of alternative splicing could partly account for increased proteomic diversity in more complex organisms[3], and an additional layer of regulation appears to confer on those genes a much greater dexterity during the development of complex organs and tissues[4]. Genes were previously thought to function in a sea of "junk" DNA, but a revolution in our understanding of nuclear processes is pointing to a critical role for these regions between genes. The proportion of nongenic regions appears to correlate with organism complexity; in yeast, 30% of the genome appears to be devoid of protein-coding potential compared to approximately 99% in mammals[5]. The ENCODE project, which aims to characterize 1% of the human genome comprehensively, has shown that over 90% of the genome is transcribed, in spite of only 1–2% of it being comprised of genes[6]. Most of these transcripts are not translated into protein and are, therefore, termed noncoding RNAs (ncRNAs). Initial debate focused on whether they represented transcriptional noise[7,8]; however, the observation that this phenomenon is shared among many species[9,10,11,12] suggests that the process is not simply a waste of cellular energy, but an important conserved mechanism for cellular function. The act of transcription has been proposed to influence the formation of subnuclear structures[13,14], the interaction of genes and regulatory elements[15,16], the mingling of chromosome territories[17], and the folding of entire chromosomes[18,19,20]. The importance of noncoding transcription is supported by a wealth of genome-wide association studies that identify intergenic regions associated with phenotypic variation and disease[21]. Indeed, increasing evidence implicates specific ncRNAs in a wide range of functional roles, suggesting that they may be the architects of an additional framework of gene regulation, mediating vast phenotypic differences with similar numbers of genes.

The nature and function of ncRNAs appears to be many and varied. A range of small (generally <50 bp) ncRNAs, including small interfering RNAs (siRNAs), microRNAs (miRNAs), and PIWI-interacting RNAs (piRNAs), have been implicated in a host of roles, such as transcriptional regulation, control of chromatin structure, heterochromatin formation, and proteomic status[22]. The role of the RNAi machinery and small ncRNAs has been extensively reviewed elsewhere[22,23]. In this review, we will focus on the role of another class of ncRNAs known as long noncoding RNAs (lncRNAs). LncRNAs vary in size from 200 bp to >100 kb, are transcribed by RNA polymerase II, and are often spliced and polyadenylated. They have been identified by a variety of means and the number of specific lncRNAs shown to influence genomic function is growing. These include lncRNAs with roles in imprinting[24], enhancer function[25,26], X chromosome inactivation[27], chromatin structure[28], and genomic rearrangements during the generation of antibody diversity[29]. They appear critical for normal development and in many cases are deregulated in diseases such as cancer[30]. In this review, we will focus on the recent exciting findings that suggest that some lncRNAs act as tethers and guides for the correct targeting of chromatin-modifying complexes to various genomic locations, with particular reference to specific genes critical to development in multicellular organisms.

## A FUNCTIONAL LINK BETWEEN RNA AND CHROMATIN

Most cells in a multicellular organism are genetically identical, yet each cell type has a characteristic pattern of gene expression that determines cellular function[31]. The transcriptional output in any given cell type is largely the result of heritable patterns of epigenetic modifications. These covalent chemical groups, which include histone methylation, acetylation, and DNA methylation, are critical for genomic structure and regulation[32]. An increasingly compelling body of work suggests that RNA plays a role in the maintenance of chromatin function. The results of biochemical experiments suggest that RNA is an integral component of chromatin, possibly playing a structural role[33,34,35]. Fluorescent *in situ* hybridization (FISH) experiments have shown that ncRNAs can coat entire chromosomes and confer epigenetic marks (discussed further below)[36]. The lncRNA NEAT1 has been shown to nucleate paraspeckles, a type of subnuclear compartment thought to be involved in RNA processing[37]. A role for RNA has also been suggested in the targeting of HP1 to heterochromatic chromatin[38].

In several gene loci, regions of lncRNA transcription have been found to overlap with domains of active chromatin modifications and a more open chromatin conformation[26,39,40,41,42]. An important question, then, is whether it is the act of transcription itself or the lncRNA working in *trans* that leads to chromatin modification, or indeed if the transcription is a result of (rather than a cause of) chromatin activation. Perturbation of these ncRNAs often results in the deregulation of chromatin modifications, suggesting that lncRNAs (or their transcription) do indeed play a role in determining chromatin structure[25,26,42,43]. The process of transcriptional elongation through a chromatinized template involves the coordinate action of many protein complexes, and includes the removal of nucleosomal proteins and redeposition behind the processing polymerase[44]. Some chromatin-modifying complexes have been found to associate with RNA polymerase, so it is conceivable that as the machinery moves through chromatin, it adds covalent modifications to histones[45,46]. Another model suggests that the lncRNAs produced through intergenic transcription bind chromatin-modifying complexes and regulate chromatin at specific loci. This is supported by the observation that many enzymes and chromatin-modifying complexes, such as DNA methyltransferases, Polycomb-group (PcG), and Trithorax-group (TrxG) proteins, appear capable of binding RNA[47,48,49,50,51]. Indeed, in many cases, it is unclear whether these enzymes are inherently capable of binding DNA or have DNA-binding partners. The use of lncRNAs as tethers and guides for the targeting of chromatin modifications is consistent with the need for a limited number of enzymes and complexes to control the epigenetic landscape of the entire genome accurately, in a cell type-specific manner, which is able to respond to the changing needs and environment of the cell. In this way lncRNAs could provide a dynamic framework in which such complexes can operate, allowing a limited gene set to be differentially regulated through development in a complex multicellular organism.

## LNCRNAS AND X CHROMOSOME REGULATION

The presence of different numbers of sex chromosomes in male and female organisms presents a special challenge to genomic regulatory mechanisms. It is important for development and survival that the expression of genes on the X chromosome is at an equivalent level in both sexes. *Drosophila* and mammals appear to have evolved opposing strategies, which share the use of lncRNAs at their heart[52]. In *Drosophila*, the single male X chromosome is transcriptionally hyperstimulated by widespread acetylation[53]. This is achieved by two lncRNAs encoded on the X chromosome, *roX1* and *roX2*, which appear to physically recruit the dosage compensation complex (DCC) to many sites along the male X chromosome, resulting in increased acetylation[54,55].

In female mammalian embryos, one of the two X chromosomes becomes epigenetically silenced[56]. This is ultimately accomplished by the lncRNA Xist, which is expressed only from the inactive X and coats the chromosome in *cis*, leading to inactivation[36,57,58]. This involves the recruitment of PcG complexes capable of conferring H3K27 trimethylation via interaction with the conserved A-repeat sequence at the 5' end of the Xist RNA[59], leading to the formation of heterochromatin and epigenetic silencing[60]. *Xist* expression is tightly regulated by the coordinate action of a group of lncRNAs situated within a 500-kb region, known as the X-inactivation center (Xic) (Fig. 1). *Tsix* is a 40-kb lncRNA expressed from the active X that overlaps with *Xist*, is transcribed in the antisense direction, and negatively regulates *Xist* in *cis*[61]. *Tsix* is itself positively regulated by an upstream regulator, *Xite*, which contains intergenic transcription potential[62]. Prior to inactivation, both X chromosomes are epigenetically similar, with some *Xist* expression detectable from both loci, but during early development, the two X chromosomes physically associate and during this period of interaction, the epigenetic asymmetry is established that eventually leads to absence of *Xist* on the active X and overexpression on the inactive X[63,64,65,66]. The contact between X chromosomes appears to be mediated by pairing elements present in a 15-kb region overlapping *Xite* and *Tsix*[66]. There is evidence suggesting that this

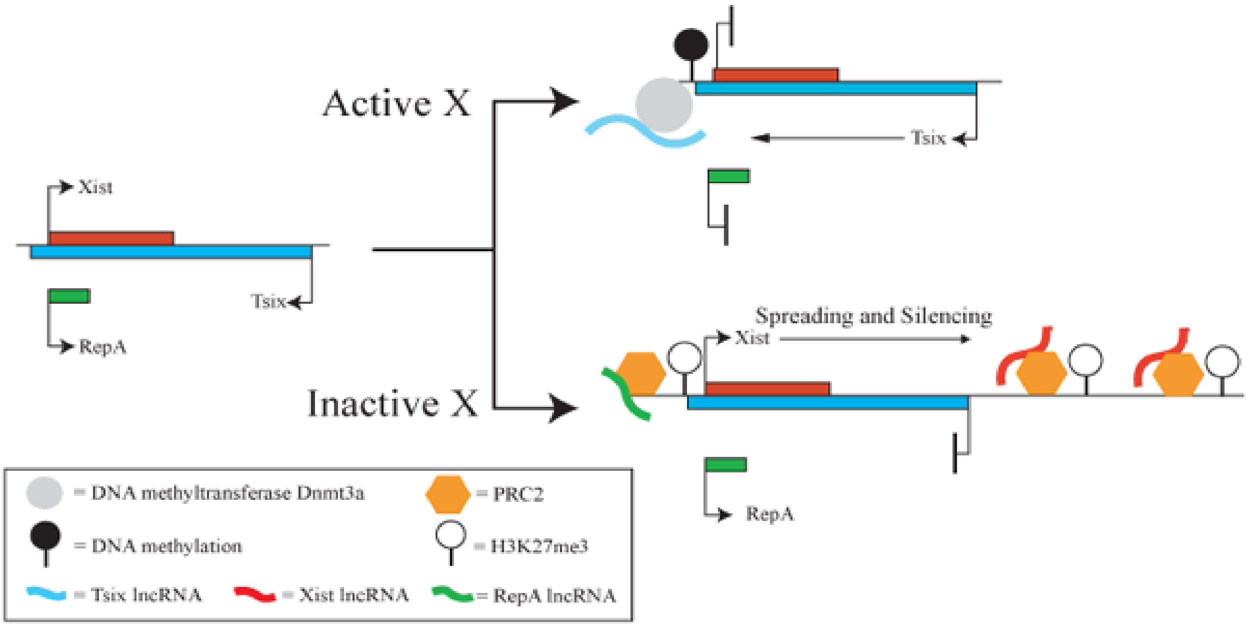

**FIGURE 1.** The role of lncRNAs in X chromosome inactivation. In the inactive X, the lncRNA RepA is transcribed and appears to recruit PRC2 to the 5' end of *Xist*, leading to the seeding of a H3K27 trimethylation heterochromatin patch. This permits production of the Xist transcript, which recruits PRC2 to various sites across the X chromosome in *cis* and mediates epigenetic silencing. In the active X, the lncRNA Tsix is produced, which may interfere with the function of RepA and prevents transcription of Xist, possibly by recruiting the DNA methyltransferase Dnmt3a to the Xist promoter region. Silencing of Xist by Tsix prevents heterochromatinization and allows one X chromosome to remain active.

pairing event may require the action of lncRNAs or the act of their transcription[67], hinting at the importance of transcription in the regulation of chromatin dynamics. Prior to inactivation, both alleles transcribe *Tsix*, which appears to keep the 40-kb region enriched for H3K4 methylation and H4 acetylation, in an apparently active state[68]. Paradoxically, this active state appears to cause repression of the Xist lncRNA in *cis*. This may be explained by the interesting finding that Tsix RNA physically associates with the DNA methyltransferase Dnmt3a at the *Xist* promoter[68], leading to DNA methylation and silencing of *Xist* only on the active X chromosome[68,69]. There is also evidence that the transcriptionally repressive DNA methylation at the *Xist* promoter may be accomplished through the RNAi pathway, following the production of small interfering RNAs from annealed Tsix and Xist transcripts[70]. In the inactive X, loss of *Tsix* expression leads to a more heterochromatic chromatin state, including increased levels of H3K27 trimethylation, loss of DNA methylation at the *Xist* promoter, and *Xist* up-regulation[68].

The paradoxical finding that *Xist* is repressed in euchromatin, but actively transcribed in heterochromatin, reflects an interesting evolutionary solution to the need to silence a chromosome using a lncRNA[27]. The accumulation of PRC2 (Polycomb Repressive Complex 2, the PcG complex responsible for H3K27 trimethylation) at the 5' end of *Xist* following *Tsix* down-regulation in the inactive X occurs before *Xist* up-regulation and is, therefore, unlikely to be mediated by the Xist RNA itself[68,71]. The recruitment of PRC2 in this case appears to be mediated by another lncRNA in the Xic, known as RepA[71]. RepA is a 1.6-kb RNA that overlaps with the 5' end of *Xist* and includes the A-repeat motif known to bind PRC2[59,71]. Upon *Tsix* down-regulation in the inactive X, RepA recruits PRC2 to create a heterochromatic "patch" in the Xic, which then seeds the activation and spread of Xist to the rest of the chromosome[27,68,71]. It is unclear how *Tsix* and RepA functionally interact, but it has been proposed that Tsix may interfere with the RepA-PRC2 interaction[27]. Regardless of the precise molecular mechanisms, the case of X chromosome dosage compensation in insects and mammals demonstrates the use of lncRNA protein complexes in the regulation of intricate epigenetic processes.

## LNCRNAS AND IMPRINTING

Autosomal imprinting is the process by which a gene is expressed from one allele, but silent at the other. Like X chromosome inactivation, imprinting appears to involve the function of lncRNAs and while there are differences between both processes, it has been suggested that they have a common evolutionary origin[72]. In placental mammals, thousands of autosomal genes and transcripts appear to be imprinted, and these are often arranged in clusters[73]. Increasingly, evidence is emerging that lncRNAs play a direct role in the regulation of various imprinted genes[24].

A cluster of imprinted genes located at the telomeric end of chromosome 7 in mice is regulated by the lncRNA Kcnq1ot1[24]. *Kcnq1ot1* is transcribed as an antisense RNA on the paternal allele from a promoter located in the tenth intron of the paternally silenced *Kcnq1* gene. Deletion of the *Kcnq1ot1* promoter, or truncation of the lncRNA by insertion of a transcription termination cassette, leads to loss of imprinting[74,75,76]. Loss of the Kcnq1ot1 RNA leads to deregulation of H3K9 dimethylation, H3K27 trimethylation, or DNA methylation at genes whose imprinting is lost[34,77,78,79]. One possible mechanism is that Kcnq1ot1 associates with the chromatin-modifying complexes, guiding them to the paternally imprinted genes. In support of this hypothesis, experiments using RNA FISH and immunostaining to visualize Kcnq1ot1 RNA, H3K9 trimethylation, and H3K27 trimethylation show that the three often overlap[80]. Furthermore, Kcnq1ot1 RNA has been found to interact with PRC2 and G9a (the enzyme responsible for H3K9 dimethylation), and the complexes appear to bind chromatin at the imprinted genes[34,81]. A region at the 5’ end of *Kcnq1ot1* appears to harbor much of the information required to modify genes epigenetically and may be sufficient to recruit imprinted genes to perinucleolar heterochromatin[82]. Interestingly, some genes in the imprinted locus appear to be silenced by subtly different mechanisms[76,79,83,84]; what role the lncRNAs play in defining these differences remains to be elucidated.

The insulin-like growth factor-2 receptor (*Igf2r)* locus provides another example of imprinting regulation through lncRNAs. In addition to *Igf2r,* the locus contains the *Slc22a2* and *Slc22a3* genes; in early mouse embryos, all three are expressed only from the maternal allele and require the action of the Air (Antisense Igf2r RNA) lncRNA for paternal imprinting[85,86,87,88]. Similar to the *Kcnq1ot1* locus, *Air* is transcribed in the antisense direction from a promoter within an imprinted gene; specifically, the second intron of the paternal *Igf2r* allele. Truncation of the Air transcript leads to biallelic expression of all three genes[87]. A modified RNA TRAP (RNA tagging and recovery of associated proteins) technique[89] was used to show that the Air transcript was associated with the promoter of at least one of the imprinted genes[86]. Furthermore, the transcript interacts with G9a and mediates H3K9 dimethylation at the imprinted gene promoters in *cis*[86], again highlighting the importance of lncRNAs in the regulation of epigenetic processes. Allelic silencing in other imprinted loci, including the *Igf2*/*H19* locus and the *Gnas* clusters, also appears to involve the function of lncRNAs[90,91].

## LNCRNAS AND THE HOX GENES

The importance of lncRNAs in controlling epigenetics is not confined to the regulation of dosage compensation. Indeed, recent results suggest that they play their part in many aspects of developmental biology. The *Hox* genes encode transcription factors containing DNA-binding homeodomains. These factors influence the expression of multiple genes during development, and are critical for determining body segmentation patterns and organ formation[92]. They are highly conserved through evolution and are often clustered[93]. The arrangement of *Hox* genes along the chromosome reflects their order of activation during development, as well as their positional expression along the anterior-posterior axis, a fascinating feature known as colinearity[93]. These genes have been extensively studied in a range of organisms, including *Drosophila* and mammals.

In *Drosophila,* the *Hox* genes are distributed in two clusters, the bithorax complex (BX-C) and the antennapedia complex (ANT-C)[94,95]. The BX-C contains three protein-coding genes, Ultrabithorax

(*Ubx*), Abdominal A (*abd-A*), and Abdominal B (*abd-b*)[94]. A number of mutations affect expression of the Ubx protein, but most do not occur in the protein-coding sequences[96], suggesting an important role for intergenic regions. A number of lncRNAs can also be detected within the *Drosophila Hox* clusters, some of which overlap with the homeotic genes and are themselves regulated spatiotemporally[97,98]. The nature and role of these lncRNAs is currently the topic of much debate. Some early experiments suggested that transcription through "maintenance elements" prevents silencing of *Ubx* and *Abd-B* by blocking the binding of negatively regulating PcG proteins and allowing recruitment of the positively regulating TrxG proteins[99,100,101,102]. More recently, it has been shown that lncRNAs produced by transcription through maintenance elements is required to recruit Ash1 (a TrxG protein capable of methylating H3K4) in *trans* to regions upstream of Ubx, leading to epigenetic activation of the Hox gene[103]. The requirement for an RNA element in this recruitment of Ash is consistent with the lack of DNA-binding capacity of this TrxG protein and the finding that RNase-treatment deregulates Ash1 binding across the genome[103]. In contrast, single-cell analyses using RNA FISH suggest a repressive role of these lncRNAs on *Ubx* through transcriptional interference[104]. Thus, while the precise role and mechanisms of lncRNAs in the *Drosophila Hox* clusters is unclear, they do appear to have an important function in epigenetic regulation of homeotic genes.

The 39 *Hox* genes in humans are distributed over four clusters, known as HOXA, HOXB, HOXC, and HOXD[93]. Recent work has shown that the clusters are epigenetically subdivided into domains of chromatin methylated at either H3K4 or H3K27, but not both[28]. Indeed, regions of transcriptional activity appear clustered in domains where H3K27 is absent[28]. Interestingly, the patterns of *Hox* gene expression in fibroblasts taken from different anatomical locations in adult humans differ from one another, and faithfully recapitulate the principles of colinearity established during embryogenesis[105,106]. This hints at a mechanism of epigenetic memory within cells responsible for body identity. Similar to the *Drosophila* loci, mammalian *Hox* clusters are punctuated by many spatiotemporally regulated lncRNAs, some of which overlap with the *Hox* genes themselves[28,107,108,109]. A recent high-resolution analysis of transcription in the human *Hox* clusters identified 231 lncRNAs expressed in a developmentally regulated manner[28]. A fascinating insight into their function was provided by the analysis of one of these lncRNAs, termed HOTAIR (*Hox* antisense intergenic RNA). HOTAIR is expressed from the HOXC cluster in a segment-dependent manner, binds to the PRC2 complex, and appears to cause widespread repression within the HOXD locus through H3K27 trimethylation (Fig. 2)[28]. Remarkably, the HOXD locus is on a separate chromosome to HOXC, suggesting that HOTAIR acts in *trans* to recruit the repressive PRC2 complex to specific genomic locations. Following the initial seeding of H3K27 trimethylation, PRC2 may itself bind the same modification and deposit further H3K27 methylation on neighboring histones[110]. Such a mechanism could reinforce the maintenance of epigenetic memory, while simultaneously providing a point for the potential integration of external stimuli with domain-wide chromatin modifications[92].

## MODELS FOR LNCRNA FUNCTION

Further examples continue to emerge of lncRNAs acting as tethers and guides for chromatin-modifying complexes. Embryonic stem cells appear to transcribe a range of lncRNAs that may be involved in maintaining pluripotency, some of which appear to associate physically with the H3K4 methyltransferase MLL1[111]. A recent method to identify potential lncRNAs, based on the chromatin signature indicative of a transcription unit, identified thousands of lncRNAs with regulatory potential[112]. RNA immunoprecipitation experiments revealed that many of these lncRNAs associate with the repressing PRC2 complex[113]. Knockdown of a selection of these PRC2-interacting lncRNAs by RNAi resulted in the transcriptional up-regulation of dozens of genes, suggesting that these lncRNAs normally function by recruiting PRC2 to multiple genes, resulting in H3K27 methylation–induced silencing[113]. Intriguingly, knockdown of the PRC2-interacting TUG1 lncRNA, previously identified as playing a role in retinal development[114], leads to the activation of many genes involved in cell-cycle regulation[113]. TUG1 itself

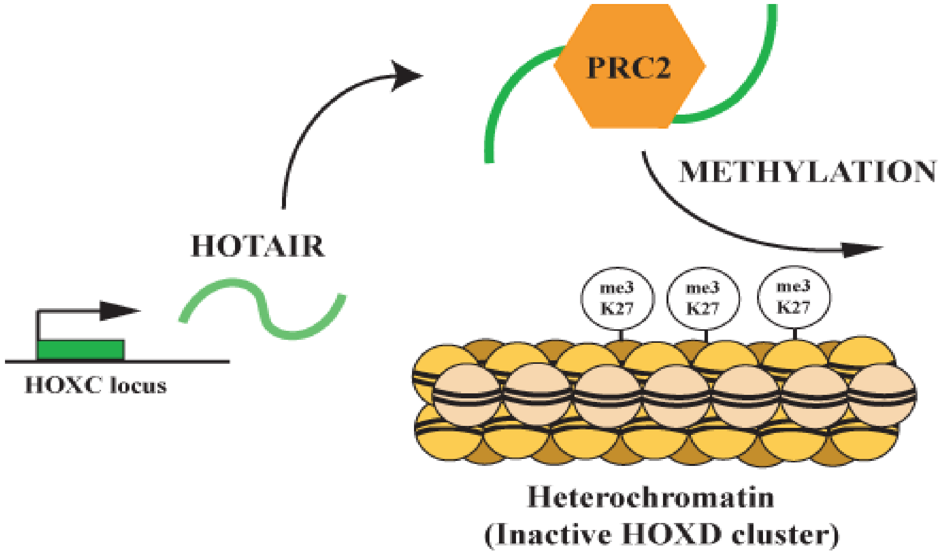

**FIGURE 2.** The role of the HOTAIR lncRNA in Hox gene regulation. HOTAIR is a lncRNA expressed from the human HOXC cluster, which binds to PRC2 and recruits the complex to the HOXD cluster, leading to H3K27 trimethylation–mediated heterochromatin formation. The two loci are on separate chromosomes, suggesting that the lncRNA works in *trans* to regulate chromatin structure.

appears to be up-regulated by the p53 protein in response to DNA damage[112], suggesting that complex networks of transcription factors, lncRNAs, and chromatin-modifying proteins interact to regulate transcriptional programs.

While the evidence for lncRNAs as *trans*-acting factors in the recruitment of chromatin-modifying complexes is compelling, there is also a plethora of data that suggest alternative mechanisms for lncRNA action. Transcriptional interference is an effect by which the act of transcribing one region can interfere with the transcription of another gene. It has been proposed by several groups that the production of one lncRNA can prevent the transcription of a gene during development[104,115,116]. It has also been suggested that transcripts that overlap in the same or opposite direction to a protein-coding gene can interfere with gene transcription by forming duplex or triplex structures with cryptic RNAs produced from the promoter[117,118]. Many genes appear to have associated antisense transcripts that may regulate their coding partner through transcriptional interference or the generation of double-stranded RNA (for examples, see Mattick[21]). Human and murine repeat-sequence transcripts appear capable of repressing transcription of specific genes during the heat shock response by associating with RNA polymerase molecules and interfering with elongation[119,120]. It seems unlikely that any one model for lncRNA function accounts for all aspects of nuclear regulation; indeed, it appears that lncRNAs have evolved a variety of means to regulate transcriptional networks, individual genes, and nuclear architecture.

## CONCLUSIONS AND UNANSWERED QUESTIONS

Many questions remain unanswered: What is the biochemical and molecular basis of lncRNAs binding to chromatin-modifying complexes and how do lncRNAs achieve target specificity? The lncRNA upstream of the *Ubx* gene in *Drosophila* appears to bind chromatin first and then recruit Ash1, the methyltransferase[103]. Indeed, treatment of *Drosophila* cells with RNase disrupts binding of Ash1 at over 100 sites in the genome[103]. The lack of high sequence conservation in lncRNAs may indicate a lack of function, but it is known that certain regions of these transcripts are conserved, and it may be these domains that mediate binding to chromatin-modifying complexes or to specific sequences[121]. The A-

repeat domain, which has been shown to mediate binding of Xist to PRC2, may represent an example of a common chromatin-lncRNA coupling mechanism[59]. Analysis of the sets of genes up-regulated when specific PRC2-interacting lncRNAs are knocked down revealed no common motif at the promoters of genes targeted by any given lncRNA[113]. The specificity of PRC2-interacting lncRNAs acting in *trans* is nevertheless tightly regulated, repressing individual genes within crowded genomic regions[113]. Given the lack of promoter-consensus sequences, what could be the basis of this choice? One possibility is that lncRNAs mediate specificity through interaction with bidirectional cryptic transcripts present at many gene promoters[122,123]. Another intriguing possibility is that target specificity is mediated not through primary sequence interactions, but by higher-order structures formed by the lncRNAs. If so, then one would predict that lncRNAs would not show sequence conservation, but instead would show regions of structural conservation, similar to the folding of enzymatic or structural proteins. In certain cases, it may be the act of transcribing a region rather than the transcript itself, which may mediate changes in chromatin structure or nuclear architecture, leading to transcriptional regulation[16,124]. Do imprinting and dosage-compensation lncRNAs, which appear to work in *cis*, and lncRNAs such as HOTAIR and TUG1, which appear to work in *trans*, represent different silencing mechanisms? Certain lncRNAs, including Air, Kcnq1ot1, and Xist, appear to spread within a confined region of the nucleus[36,86,125], prompting suggestions that they nucleate repressive nuclear domains into which genes are recruited[86,125]. This is consistent with findings that genes that initially escape imprinting or *Xist*-mediated inactivation are found outside of the clouds of lncRNA, but then appear to move into the repressive lncRNA domain upon silencing[86,125,126,127]. As chromosome territories can only diffuse within a constrained region of the nucleus[128], production of an imprinted lncRNA at a transcription factory could nucleate the formation of a repressive domain that is only capable of recruiting genes from the same constrained region of the nucleus, preventing inappropriate inactivation of both loci. Another model for lncRNA action proposes that the 5’ end of a lncRNA, which in some cases appears to be the “business end”, guides chromatin-modifying complexes to the regions close to the site of transcription (either in the primary sequence or through physical looping to more distant genomic locations), while the lncRNA is still tethered to chromatin by continued transcription of the 3’ end[27]. Whether lncRNAs such as HOTAIR also work by establishing nuclear domains into which HOXD regions are recruited and repressed remains to be established.

While the precise mechanisms remain to be determined, lncRNAs are emerging as key regulators of the epigenome, influencing transcriptional networks and multicellular development. Fascinatingly, this layer of lncRNA regulation may couple chromatin to the factors that control its structure and function. Uncovering the extent to which lncRNAs regulate development and the nuances of their molecular mechanisms will be an exciting process, and may one day allow us to interfere with epigenetic processes in a targeted manner in the treatment of diseases such as cancer.

## ACKNOWLEDGMENTS

DRFC is supported by a grant from the British Society for Haematology, DPC is supported by a grant from the Dunhill Medical Trust, RCP is supported by a grant from Action Medical Research, and DT is supported by an FPI fellowship from the Spanish Government (MICINN).

## References

1. Stein, L., Bao, Z., Blasiar, D., Blumenthal, T., Brent, M., Chen, N., Chinwalla, A., Clarke, L., Clee, C., Coghlan, A., Coulson, A., D'Eustachio, P., Fitch, D., Fulton, L., Fulton, R., Griffiths-Jones, S., Harris, T., Hillier, L., Kamath, R., Kuwabara, P., Mardis, E., Marra, M., Miner, T., Minx, P., Mullikin, J., Plumb, R., Rogers, J., Schein, J., Sohrmann, M., Spieth, J., Stajich, J., Wei, C., Willey, D., Wilson, R., Durbin, R., and Waterston, R. (2003) The genome sequence of Caenorhabditis briggsae: a platform for comparative genomics. *PLoS Biol.* **1,** E45.
2. Goodstadt, L. and Ponting, C. (2006) Phylogenetic reconstruction of orthology, paralogy, and conserved synteny for dog and human. *PLoS Comput. Biol.* **2,** e133.

3. Graveley, B. (2001) Alternative splicing: increasing diversity in the proteomic world. *Trends Genet.* **17,** 100–107.
4. Costa, F. (2008) Non-coding RNAs, epigenetics and complexity. *Gene* **410,** 9–17.
5. Taft, R., Pheasant, M., and Mattick, J. (2007) The relationship between non-protein-coding DNA and eukaryotic complexity. *Bioessays* **29,** 288–299.
6. Birney, E., Stamatoyannopoulos, J., Dutta, A., Guigó, R., Gingeras, T., Margulies, E., Weng, Z., Snyder, M., Dermitzakis, E., Thurman, R., Kuehn, M., Taylor, C., Neph, S., Koch, C., Asthana, S., Malhotra, A., Adzhubei, I., Greenbaum, J., Andrews, R., Flicek, P., Boyle, P., Cao, H., Carter, N., Clelland, G., Davis, S., Day, N., Dhami, P., Dillon, S., Dorschner, M., Fiegler, H., Giresi, P., Goldy, J., Hawrylycz, M., Haydock, A., Humbert, R., James, K., Johnson, B., Johnson, E., Frum, T., Rosenzweig, E., Karnani, N., Lee, K., Lefebvre, G., Navas, P., Neri, F., Parker, S., Sabo, P., Sandstrom, R., Shafer, A., Vetrie, D., Weaver, M., Wilcox, S., Yu, M., Collins, F., Dekker, J., Lieb, J., Tullius, T., Crawford, G., Sunyaev, S., Noble, W., Dunham, I., Denoeud, F., Reymond, A., Kapranov, P., Rozowsky, J., Zheng, D., Castelo, R., Frankish, A., Harrow, J., Ghosh, S., Sandelin, A., Hofacker, I., Baertsch, R., Keefe, D., Dike, S., Cheng, J., Hirsch, H., Sekinger, E., Lagarde, J., Abril, J., Shahab, A., Flamm, C., Fried, C., Hackermüller, J., Hertel, J., Lindemeyer, M., Missal, K., Tanzer, A., Washietl, S., Korbel, J., Emanuelsson, O., Pedersen, J., Holroyd, N., Taylor, R., Swarbreck, D., Matthews, N., Dickson, M., Thomas, D., Weirauch, M., Gilbert, J., Drenkow, J., Bell, I., Zhao, X., Srinivasan, K., Sung, W., Ooi, H., Chiu, K., Foissac, S., Alioto, T., Brent, M., Pachter, L., Tress, M., Valencia, A., Choo, S., Choo, C., Ucla, C., Manzano, C., Wyss, C., Cheung, E., Clark, T., Brown, J., Ganesh, M., Patel, S., Tammana, H., Chrast, J., Henrichsen, C., Kai, C., Kawai, J., Nagalakshmi, U., Wu, J., Lian, Z., Lian, J., Newburger, P., Zhang, X., Bickel, P., Mattick, J., Carninci, P., Hayashizaki, Y., Weissman, S., Hubbard, T., Myers, R., Rogers, J., Stadler, P., Lowe, T., Wei, C., Ruan, Y., Struhl, K., Gerstein, M., Antonarakis, S., Fu, Y., Green, E., Karaöz, U., Siepel, A., Taylor, J., Liefer, L., Wetterstrand, K., Good, P., Feingold, E., Guyer, M., Cooper, G., Asimenos, G., Dewey, C., Hou, M., Nikolaev, S., Montoya-Burgos, J., Löytynoja, A., Whelan, S., Pardi, F., Massingham, T., Huang, H., Zhang, N., Holmes, I., Mullikin, J., Ureta-Vidal, A., Paten, B., Seringhaus, M., Church, D., Rosenbloom, K., Kent, W., Stone, E., Batzoglou, S., Goldman, N., Hardison, R., Haussler, D., Miller, W., Sidow, A., Trinklein, N., Zhang, Z., Barrera, L., Stuart, R., King, D., Ameur, A., Enroth, S., Bieda, M., Kim, J., Bhinge, A., Jiang, N., Liu, J., Yao, F., Vega, V., Lee, C., Ng, P., Yang, A., Moqtaderi, Z., Zhu, Z., Xu, X., Squazzo, S., Oberley, M., Inman, D., Singer, M., Richmond, T., Munn, K., Rada-Iglesias, A., Wallerman, O., Komorowski, J., Fowler, J., Couttet, P., Bruce, A., Dovey, O., Ellis, P., Langford, C., Nix, D., Euskirchen, G., Hartman, S., Urban, A., Kraus, P., Van Calcar, S., Heintzman, N., Kim, T., Wang, K., Qu, C., Hon, G., Luna, R., Glass, C., Rosenfeld, M., Aldred, S., Cooper, S., Halees, A., Lin, J., Shulha, H., Xu, M., Haidar, J., Yu, Y., Iyer, V., Green, R., Wadelius, C., Farnham, P., Ren, B., Harte, R., Hinrichs, A., Trumbower, H., Clawson, H., Hillman-Jackson, J., Zweig, A., Smith, K., Thakkapallayil, A., Barber, G., Kuhn, R., Karolchik, D., Armengol, L., Bird, C., de Bakker, P., Kern, A., Lopez-Bigas, N., Martin, J., Stranger, B., Woodroffe, A., Davydov, E., Dimas, A., Eyras, E., Hallgrímsdóttir, I., Huppert, J., Zody, M., Abecasis, G., Estivill, X., Bouffard, G., Guan, X., Hansen, N., Idol, J., Maduro, V., Maskeri, B., McDowell, J., Park, M., Thomas, P., Young, A., Blakesley, R., Muzny, D., Sodergren, E., Wheeler, D., Worley, K., Jiang, H., Weinstock, G., Gibbs, R., Graves, T., Fulton, R., Mardis, E., Wilson, R., Clamp, M., Cuff, J., Gnerre, S., Jaffe, D., Chang, J., Lindblad-Toh, K., Lander, E., Koriabine, M., Nefedov, M., Osoegawa, K., Yoshinaga, Y., Zhu, B., and de Jong, P. (2007) Identification and analysis of functional elements in 1% of the human genome by the ENCODE pilot project. *Nature* **447,** 799–816.
7. Ponjavic, J., Ponting, C., and Lunter, G. (2007) Functionality or transcriptional noise? Evidence for selection within long noncoding RNAs. *Genome Res*. **17,** 556–565.
8. Struhl, K. (2007) Transcriptional noise and the fidelity of initiation by RNA polymerase II. *Nat. Struct. Mol. Biol.* **14,** 103–105.
9. Kapranov, P., Willingham, A., and Gingeras, T. (2007) Genome-wide transcription and the implications for genomic organization. *Nat. Rev. Genet.* **8,** 413–423.
10. Kapranov, P., Cheng, J., Dike, S., Nix, D., Duttagupta, R., Willingham, A., Stadler, P., Hertel, J., Hackermüller, J., Hofacker, I., Bell, I., Cheung, E., Drenkow, J., Dumais, E., Patel, S., Helt, G., Ganesh, M., Ghosh, S., Piccolboni, A., Sementchenko, V., Tammana, H., and Gingeras, T. (2007) RNA maps reveal new RNA classes and a possible function for pervasive transcription. *Science* **316,** 1484–1488.
11. He, H., Wang, J., Liu, T., Liu, X., Li, T., Wang, Y., Qian, Z., Zheng, H., Zhu, X., Wu, T., Shi, B., Deng, W., Zhou, W., Skogerbø, G., and Chen, R. (2007) Mapping the C. elegans noncoding transcriptome with a whole-genome tiling microarray. *Genome Res.* **17,** 1471–1477.
12. Manak, J., Dike, S., Sementchenko, V., Kapranov, P., Biemar, F., Long, J., Cheng, J., Bell, I., Ghosh, S., Piccolboni, A., and Gingeras, T. (2006) Biological function of unannotated transcription during the early development of Drosophila melanogaster. *Nat. Genet.* **38,** 1151–1158.
13. Eskiw, C., Rapp, A., Carter, D., and Cook, P. (2008) RNA polymerase II activity is located on the surface of protein-rich transcription factories. *J. Cell Sci.* **121,** 1999–2007.
14. Osborne, C., Chakalova, L., Brown, K., Carter, D., Horton, A., Debrand, E., Goyenechea, B., Mitchell, J., Lopes, S., Reik, W., and Fraser, P. (2004) Active genes dynamically colocalize to shared sites of ongoing transcription. *Nat. Genet.* **36,** 1065–1071.
15. Carter, D., Chakalova, L., Osborne, C., Dai, Y., and Fraser, P. (2002) Long-range chromatin regulatory interactions in vivo. *Nat. Genet*. **32,** 623–626.
16. Cook, P. (2003) Nongenic transcription, gene regulation and action at a distance. *J. Cell Sci.* **116,** 4483–4491.
17. Branco, M. and Pombo, A. (2006) Intermingling of chromosome territories in interphase suggests role in translocations and transcription-dependent associations. *PLoS Biol.* **4,** e138.
18. Simonis, M., Klous, P., Splinter, E., Moshkin, Y., Willemsen, R., de Wit, E., van Steensel, B., and de Laat, W. (2006) Nuclear organization of active and inactive chromatin domains uncovered by chromosome conformation capture-on-chip (4C). *Nat. Genet.* **38,** 1348–1354.
19. Carter, D., Eskiw, C., and Cook, P. (2008) Transcription factories. *Biochem. Soc. Trans.* **36,** 585–589.
20. Sexton, T., Umlauf, D., Kurukuti, S., and Fraser, P. (2007) The role of transcription factories in large-scale structure and dynamics of interphase chromatin. *Semin. Cell Dev. Biol.* **18,** 691–697.
21. Mattick, J. (2009) The genetic signatures of noncoding RNAs. *PLoS Genet.* **5,** e1000459.
22. Costa, F. (2007) Non-coding RNAs: lost in translation? *Gene* **386,** 1–10.
23. Siomi, H. and Siomi, M. (2009) On the road to reading the RNA-interference code. *Nature* **457,** 396–404.
24. Nagano, T. and Fraser, P. (2009) Emerging similarities in epigenetic gene silencing by long noncoding RNAs. *Mamm. Genome*. [Epub ahead of print]
25. Kim, A., Zhao, H., Ifrim, I., and Dean, A. (2007) Beta-globin intergenic transcription and histone acetylation dependent on an enhancer. *Mol. Cell. Biol.* **27,** 2980–2986.
26. Ho, Y., Elefant, F., Liebhaber, S., and Cooke, N. (2006) Locus control region transcription plays an active role in long-range gene activation. *Mol. Cell* **23,** 365–375.
27. Lee, J. (2009) Lessons from X-chromosome inactivation: long ncRNA as guides and tethers to the epigenome. *Genes Dev.* **23,** 1831–1842.
28. Rinn, J., Kertesz, M., Wang, J., Squazzo, S., Xu, X., Brugmann, S., Goodnough, L., Helms, J., Farnham, P., Segal, E., and Chang, H. (2007) Functional demarcation of active and silent chromatin domains in human HOX loci by noncoding RNAs. *Cell* **129,** 1311–1323.
29. Krangel, M. (2007) T cell development: better living through chromatin. *Nat. Immunol.* **8,** 687–694.
30. Perez, D., Hoage, T., Pritchett, J., Ducharme-Smith, A., Halling, M., Ganapathiraju, S., Streng, P., and Smith, D. (2008) Long, abundantly expressed non-coding transcripts are altered in cancer. *Hum. Mol. Genet*. **17,** 642–655.
31. Bernstein, B., Meissner, A., and Lander, E. (2007) The mammalian epigenome. *Cell* **128,** 669–681.
32. Kouzarides, T. (2007) Chromatin modifications and their function. *Cell* **128,** 693–705.
33. Bernstein, E., Duncan, E., Masui, O., Gil, J., Heard, E., and Allis, C. (2006) Mouse polycomb proteins bind differentially to methylated histone H3 and RNA and are enriched in facultative heterochromatin. *Mol. Cell. Biol.* **26,** 2560–2569.
34. Pandey, R., Mondal, T., Mohammad, F., Enroth, S., Redrup, L., Komorowski, J., Nagano, T., Mancini-Dinardo, D., and Kanduri, C. (2008) Kcnq1ot1 antisense noncoding RNA mediates lineage-specific transcriptional silencing through chromatin-level regulation. *Mol. Cell* **32,** 232–246.
35. Rodríguez-Campos, A. and Azorín, F. (2007) RNA is an integral component of chromatin that contributes to its structural organization. *PLoS One* **2,** e1182.
36. Clemson, C., McNeil, J., Willard, H., and Lawrence, J. (1996) XIST RNA paints the inactive X chromosome at interphase: evidence for a novel RNA involved in nuclear/chromosome structure. *J. Cell Biol.* **132,** 259–275.
37. Clemson, C., Hutchinson, J., Sara, S., Ensminger, A., Fox, A., Chess, A., and Lawrence, J. (2009) An architectural role for a nuclear noncoding RNA: NEAT1 RNA is essential for the structure of paraspeckles. *Mol. Cell* **33,** 717–726.
38. Muchardt, C., Guilleme, M., Seeler, J., Trouche, D., Dejean, A., and Yaniv, M. (2002) Coordinated methyl and RNA binding is required for heterochromatin localization of mammalian HP1alpha. *EMBO Rep.* **3,** 975–981.
39. Chowdhury, M., Forouhi, O., Dayal, S., McCloskey, N., Gould, H., Felsenfeld, G., and Fear, D. (2008) Analysis of intergenic transcription and histone modification across the human immunoglobulin heavy-chain locus. *Proc. Natl. Acad. Sci. U. S. A.* **105,** 15872–15877.
40. Gribnau, J., Diderich, K., Pruzina, S., Calzolari, R., and Fraser, P. (2000) Intergenic transcription and developmental remodeling of chromatin subdomains in the human beta-globin locus. *Mol. Cell* **5,** 377–386.
41. Miles, J., Mitchell, J., Chakalova, L., Goyenechea, B., Osborne, C., O'Neill, L., Tanimoto, K., Engel, J., and Fraser, P. (2007) Intergenic transcription, cell-cycle and the developmentally regulated epigenetic profile of the human beta-globin locus. *PLoS One* **2,** e630.
42. Abarrategui, I. and Krangel, M. (2006) Regulation of T cell receptor-alpha gene recombination by transcription. *Nat. Immunol.* **7,** 1109–1115.
43. Abarrategui, I. and Krangel, M. (2007) Noncoding transcription controls downstream promoters to regulate T-cell receptor alpha recombination. *EMBO J.* **26,** 4380–4390.
44. Belotserkovskaya, R., Oh, S., Bondarenko, V., Orphanides, G., Studitsky, V., and Reinberg, D. (2003) FACT facilitates transcription-dependent nucleosome alteration. *Science* **301,** 1090–1093.
45. Travers, A. (1999) Chromatin modification by DNA tracking. *Proc. Natl. Acad. Sci. U. S. A.* **96,** 13634–13637.
46. Pavri, R., Zhu, B., Li, G., Trojer, P., Mandal, S., Shilatifard, A., and Reinberg, D. (2006) Histone H2B monoubiquitination functions cooperatively with FACT to regulate elongation by RNA polymerase II. *Cell* **125,** 703–717.
47. Mattick, J. (2001) Non-coding RNAs: the architects of eukaryotic complexity. *EMBO Rep.* **2,** 986–991.

48. Cassiday, L.A. and Maher, L.J., 3rd (2002) Having it both ways: transcription factors that bind DNA and RNA. *Nucleic Acids Res.* **30,** 4118–4126.
49. Jeffery, L. and Nakielny, S. (2004) Components of the DNA methylation system of chromatin control are RNA-binding proteins. *J. Biol. Chem.* **279,** 49479–49487.
50. Shimojo, H., Sano, N., Moriwaki, Y., Okuda, M., Horikoshi, M., and Nishimura, Y. (2008) Novel structural and functional mode of a knot essential for RNA binding activity of the Esa1 presumed chromodomain. *J. Mol. Biol.* **378,** 987–1001.
51. Bernstein, E. and Allis, C. (2005) RNA meets chromatin. *Genes Dev.* **19,** 1635–1655.
52. Angelopoulou, R., Lavranos, G., and Manolakou, P. (2008) Regulatory RNAs and chromatin modification in dosage compensation: a continuous path from flies to humans? *Reprod. Biol. Endocrinol.* **6,** 12.
53. Deng, X. and Meller, V. (2006) Non-coding RNA in fly dosage compensation. *Trends Biochem. Sci.* **31,** 526–532.
54. Kotlikova, I., Demakova, O., Semeshin, V., Shloma, V., Boldyreva, L., Kuroda, M., and Zhimulev, I. (2006) The Drosophila dosage compensation complex binds to polytene chromosomes independently of developmental changes in transcription. *Genetics* **172,** 963–974.
55. Legube, G., McWeeney, S., Lercher, M., and Akhtar, A. (2006) X-chromosome-wide profiling of MSL-1 distribution and dosage compensation in Drosophila. *Genes Dev.* **20,** 871–883.
56. Payer, B. and Lee, J. (2008) X chromosome dosage compensation: how mammals keep the balance. *Annu. Rev. Genet*. **42,** 733–772.
57. Brockdorff, N., Ashworth, A., Kay, G., McCabe, V., Norris, D., Cooper, P., Swift, S., and Rastan, S. (1992) The product of the mouse Xist gene is a 15 kb inactive X-specific transcript containing no conserved ORF and located in the nucleus. *Cell* **71,** 515–526.
58. Penny, G., Kay, G., Sheardown, S., Rastan, S., and Brockdorff, N. (1996) Requirement for Xist in X chromosome inactivation. *Nature* **379,** 131–137.
59. Wutz, A., Rasmussen, T., and Jaenisch, R. (2002) Chromosomal silencing and localization are mediated by different domains of Xist RNA. *Nat. Genet.* **30,** 167–174.
60. Plath, K., Fang, J., Mlynarczyk-Evans, S., Cao, R., Worringer, K., Wang, H., de la Cruz, C., Otte, A., Panning, B., and Zhang, Y. (2003) Role of histone H3 lysine 27 methylation in X inactivation. *Science* **300,** 131–135.
61. Lee, J. and Lu, N. (1999) Targeted mutagenesis of Tsix leads to nonrandom X inactivation. *Cell* **99,** 47–57.
62. Ogawa, Y. and Lee, J. (2003) Xite, X-inactivation intergenic transcription elements that regulate the probability of choice. *Mol. Cell* **11,** 731–743.
63. Anguera, M., Sun, B., Xu, N., and Lee, J. (2006) X-chromosome kiss and tell: how the Xs go their separate ways. *Cold Spring Harb. Symp. Quant. Biol.* **71,** 429–437.
64. Bacher, C., Guggiari, M., Brors, B., Augui, S., Clerc, P., Avner, P., Eils, R., and Heard, E. (2006) Transient colocalization of X-inactivation centres accompanies the initiation of X inactivation. *Nat. Cell Biol.* **8,** 293–299.
65. Carrel, L. (2006) Molecular biology. "X"-rated chromosomal rendezvous. *Science* **311,** 1107–1109.
66. Xu, N., Tsai, C., and Lee, J. (2006) Transient homologous chromosome pairing marks the onset of X inactivation. *Science* **311,** 1149–1152.
67. Xu, N., Donohoe, M., Silva, S., and Lee, J. (2007) Evidence that homologous X-chromosome pairing requires transcription and Ctcf protein. *Nat. Genet.* **39,** 1390–1396.
68. Sun, B., Deaton, A., and Lee, J. (2006) A transient heterochromatic state in Xist preempts X inactivation choice without RNA stabilization. *Mol. Cell* **21**, 617–628.
69. Sado, T., Wang, Z., Sasaki, H., and Li, E. (2001) Regulation of imprinted X-chromosome inactivation in mice by Tsix. *Development* **128,** 1275–1286.
70. Ogawa, Y., Sun, B., and Lee, J. (2008) Intersection of the RNA interference and X-inactivation pathways. *Science* **320,** 1336–1341.
71. Zhao, J., Sun, B., Erwin, J., Song, J., and Lee, J. (2008) Polycomb proteins targeted by a short repeat RNA to the mouse X chromosome. *Science* **322,** 750–756.
72. Reik, W. and Lewis, A. (2005) Co-evolution of X-chromosome inactivation and imprinting in mammals. *Nat. Rev. Genet.* **6,** 403–410.
73. Verona, R., Mann, M., and Bartolomei, M. (2003) Genomic imprinting: intricacies of epigenetic regulation in clusters. *Annu. Rev. Cell Dev. Biol.* **19,** 237–259.
74. Thakur, N., Tiwari, V., Thomassin, H., Pandey, R., Kanduri, M., Göndör, A., Grange, T., Ohlsson, R., and Kanduri, C. (2004) An antisense RNA regulates the bidirectional silencing property of the Kcnq1 imprinting control region. *Mol. Cell. Biol.* **24,** 7855–7862.
75. Mancini-Dinardo, D., Steele, S., Levorse, J., Ingram, R., and Tilghman, S. (2006) Elongation of the Kcnq1ot1 transcript is required for genomic imprinting of neighboring genes. *Genes Dev.* **20,** 1268–1282.
76. Shin, J., Fitzpatrick, G., and Higgins, M. (2008) Two distinct mechanisms of silencing by the KvDMR1 imprinting control region. *EMBO J.* **27,** 168–178.
77. Fitzpatrick, G., Soloway, P., and Higgins, M. (2002) Regional loss of imprinting and growth deficiency in mice with a targeted deletion of KvDMR1. *Nat. Genet.* **32,** 426–431.
78. Umlauf, D., Goto, Y., Cao, R., Cerqueira, F., Wagschal, A., Zhang, Y., and Feil, R. (2004) Imprinting along the Kcnq1 domain on mouse chromosome 7 involves repressive histone methylation and recruitment of Polycomb group complexes. *Nat. Genet*. **36**, 1296–1300.
79. Lewis, A., Mitsuya, K., Umlauf, D., Smith, P., Dean, W., Walter, J., Higgins, M., Feil, R., and Reik, W. (2004) Imprinting on distal chromosome 7 in the placenta involves repressive histone methylation independent of DNA methylation. *Nat. Genet.* **36,** 1291–1295.
80. Terranova, R., Yokobayashi, S., Stadler, M., Otte, A., van Lohuizen, M., Orkin, S., and Peters, A. (2008) Polycomb group proteins Ezh2 and Rnf2 direct genomic contraction and imprinted repression in early mouse embryos. *Dev. Cell* **15,** 668–679.
81. Murakami, K., Oshimura, M., and Kugoh, H. (2007) Suggestive evidence for chromosomal localization of non-coding RNA from imprinted LIT1. *J. Hum. Genet.* **52,** 926–933.
82. Mohammad, F., Pandey, R., Nagano, T., Chakalova, L., Mondal, T., Fraser, P., and Kanduri, C. (2008) Kcnq1ot1/Lit1 noncoding RNA mediates transcriptional silencing by targeting to the perinucleolar region. *Mol. Cell. Biol.* **28,** 3713–3728.
83. Wagschal, A., Sutherland, H., Woodfine, K., Henckel, A., Chebli, K., Schulz, R., Oakey, R., Bickmore, W., and Feil, R. (2008) G9a histone methyltransferase contributes to imprinting in the mouse placenta. *Mol. Cell. Biol.* **28,** 1104–1113.
84. Mager, J., Montgomery, N., de Villena, F., and Magnuson, T. (2003) Genome imprinting regulated by the mouse Polycomb group protein Eed. *Nat. Genet*. **33,** 502–507.
85. Lyle, R., Watanabe, D., te Vruchte, D., Lerchner, W., Smrzka, O., Wutz, A., Schageman, J., Hahner, L., Davies, C., and Barlow, D. (2000) The imprinted antisense RNA at the Igf2r locus overlaps but does not imprint Mas1. *Nat. Genet*. **25,** 19–21.
86. Nagano, T., Mitchell, J., Sanz, L., Pauler, F., Ferguson-Smith, A., Feil, R., and Fraser, P. (2008) The Air noncoding RNA epigenetically silences transcription by targeting G9a to chromatin. *Science* **322,** 1717–1720.
87. Sleutels, F., Zwart, R., and Barlow, D. (2002) The non-coding Air RNA is required for silencing autosomal imprinted genes. *Nature* **415,** 810–813.
88. Zwart, R., Sleutels, F., Wutz, A., Schinkel, A., and Barlow, D. (2001) Bidirectional action of the Igf2r imprint control element on upstream and downstream imprinted genes. *Genes Dev*. **15,** 2361–2366.
89. Chakalova, L., Carter, D., and Fraser, P. (2004) RNA fluorescence in situ hybridization tagging and recovery of associated proteins to analyze in vivo chromatin interactions. *Methods Enzymol.* **375,** 479–493.
90. Schoenfelder, S., Smits, G., Fraser, P., Reik, W., and Paro, R. (2007) Non-coding transcripts in the H19 imprinting control region mediate gene silencing in transgenic Drosophila. *EMBO Rep.* **8,** 1068–1073.
91. Chotalia, M., Smallwood, S., Ruf, N., Dawson, C., Lucifero, D., Frontera, M., James, K., Dean, W., and Kelsey, G. (2009) Transcription is required for establishment of germline methylation marks at imprinted genes. *Genes Dev*. **23,** 105–117.
92. Wang, K., Helms, J., and Chang, H. (2009) Regeneration, repair and remembering identity: the three Rs of Hox gene expression. *Trends Cell Biol.* **19,** 268–275.
93. Lemons, D. and McGinnis, W. (2006) Genomic evolution of Hox gene clusters. *Science* **313,** 1918–1922.
94. Maeda, R. and Karch, F. (2006) The ABC of the BX-C: the bithorax complex explained. *Development* **133,** 1413–1422.
95. Brock, H., Hodgson, J., Petruk, S., and Mazo, A. (2009) Regulatory noncoding RNAs at Hox loci. *Biochem. Cell Biol.* **87,** 27–34.
96. Hogness, D., Lipshitz, H., Beachy, P., Peattie, D., Saint, R., Goldschmidt-Clermont, M., Harte, P., Gavis, E., and Helfand, S. (1985) Regulation and products of the Ubx domain of the bithorax complex. *Cold Spring Harb. Symp. Quant. Biol.* **50,** 181–194.
97. Bae, E., Calhoun, V., Levine, M., Lewis, E., and Drewell, R. (2002) Characterization of the intergenic RNA profile at abdominal-A and Abdominal-B in the Drosophila bithorax complex. *Proc. Natl. Acad. Sci. U. S. A.* **99,** 16847–16852.
98. Drewell, R., Bae, E., Burr, J., and Lewis, E. (2002) Transcription defines the embryonic domains of cis-regulatory activity at the Drosophila bithorax complex. *Proc. Natl. Acad. Sci. U. S. A.* **99,** 16853–16858.
99. Bender, W. and Fitzgerald, D. (2002) Transcription activates repressed domains in the Drosophila bithorax complex. *Development* **129,** 4923–4930.
100. Hogga, I. and Karch, F. (2002) Transcription through the iab-7 cis-regulatory domain of the bithorax complex interferes with maintenance of Polycomb-mediated silencing. *Development* **129,** 4915–4922.
101. Schmitt, S., Prestel, M., and Paro, R. (2005) Intergenic transcription through a polycomb group response element counteracts silencing. *Genes Dev.* **19,** 697–708.
102. Akbari, O., Bousum, A., Bae, E., and Drewell, R. (2006) Unraveling cis-regulatory mechanisms at the abdominal-A and Abdominal-B genes in the Drosophila bithorax complex. *Dev. Biol.* **293,** 294–304.
103. Sanchez-Elsner, T., Gou, D., Kremmer, E., and Sauer, F. (2006) Noncoding RNAs of trithorax response elements recruit Drosophila Ash1 to Ultrabithorax. *Science* **311,** 1118–1123.
104. Petruk, S., Sedkov, Y., Riley, K., Hodgson, J., Schweisguth, F., Hirose, S., Jaynes, J., Brock, H., and Mazo, A. (2006) Transcription of bxd noncoding RNAs promoted by trithorax represses Ubx in cis by transcriptional interference. *Cell* **127,** 1209–1221.
105. Rinn, J., Bondre, C., Gladstone, H., Brown, P., and Chang, H. (2006) Anatomic demarcation by positional variation in fibroblast gene expression programs. *PLoS Genet.* **2,** e119.
106. Rinn, J., Wang, J., Allen, N., Brugmann, S., Mikels, A., Liu, H., Ridky, T., Stadler, H., Nusse, R., Helms, J., and Chang, H. (2008) A dermal HOX transcriptional program regulates site-specific epidermal fate. *Genes Dev.* **22,** 303–307.

107. Sessa, L., Breiling, A., Lavorgna, G., Silvestri, L., Casari, G., and Orlando, V. (2007) Noncoding RNA synthesis and loss of Polycomb group repression accompanies the colinear activation of the human HOXA cluster. *RNA* **13,** 223–239.
108. Katayama, S., Tomaru, Y., Kasukawa, T., Waki, K., Nakanishi, M., Nakamura, M., Nishida, H., Yap, C., Suzuki, M., Kawai, J., Suzuki, H., Carninci, P., Hayashizaki, Y., Wells, C., Frith, M., Ravasi, T., Pang, K., Hallinan, J., Mattick, J., Hume, D., Lipovich, L., Batalov, S., Engström, P., Mizuno, Y., Faghihi, M., Sandelin, A., Chalk, A., Mottagui-Tabar, S., Liang, Z., Lenhard, B., and Wahlestedt, C. (2005) Antisense transcription in the mammalian transcriptome. *Science* **309,** 1564–1566.
109. Mainguy, G., Koster, J., Woltering, J., Jansen, H., and Durston, A. (2007) Extensive polycistronism and antisense transcription in the mammalian Hox clusters. *PLoS One* **2,** e356.
110. Margueron, R., Justin, N., Ohno, K., Sharpe, M.L., Son, J., Drury, W.J., 3rd, Voigt, P., Martin, S.R, Taylor, W.R, De Marco, V., Pirrotta, V., Reinberg, D., and Gamblin, S.J. (2009) Role of the polycomb protein EED in the propagation of repressive histone marks. *Nature* **461(7265),** 762–767.
111. Dinger, M., Amaral, P., Mercer, T., Pang, K., Bruce, S., Gardiner, B., Askarian-Amiri, M., Ru, K., Soldà, G., Simons, C., Sunkin, S., Crowe, M., Grimmond, S., Perkins, A., and Mattick, J. (2008) Long noncoding RNAs in mouse embryonic stem cell pluripotency and differentiation. *Genome Res.* **18,** 1433–1445.
112. Guttman, M., Amit, I., Garber, M., French, C., Lin, M., Feldser, D., Huarte, M., Zuk, O., Carey, B., Cassady, J., Cabili, M., Jaenisch, R., Mikkelsen, T., Jacks, T., Hacohen, N., Bernstein, B., Kellis, M., Regev, A., Rinn, J., and Lander, E. (2009) Chromatin signature reveals over a thousand highly conserved large non-coding RNAs in mammals. *Nature* **458,** 223–227.
113. Khalil, A., Guttman, M., Huarte, M., Garber, M., Raj, A., Rivea Morales, D., Thomas, K., Presser, A., Bernstein, B., van Oudenaarden, A., Regev, A., Lander, E., and Rinn, J. (2009) Many human large intergenic noncoding RNAs associate with chromatin-modifying complexes and affect gene expression. *Proc. Natl. Acad. Sci. U. S. A.* **106,** 11667–11672.
114. Young, T., Matsuda, T., and Cepko, C. (2005) The noncoding RNA taurine upregulated gene 1 is required for differentiation of the murine retina. *Curr. Biol.* **15,** 501–512.
115. Martens, J., Laprade, L., and Winston, F. (2004) Intergenic transcription is required to repress the Saccharomyces cerevisiae SER3 gene. *Nature* **429,** 571–574.
116. Hongay, C., Grisafi, P., Galitski, T., and Fink, G. (2006) Antisense transcription controls cell fate in Saccharomyces cerevisiae. *Cell* **127,** 735–745.
117. Martianov, I., Ramadass, A., Serra Barros, A., Chow, N., and Akoulitchev, A. (2007) Repression of the human dihydrofolate reductase gene by a non-coding interfering transcript. *Nature* **445,** 666–670.
118. Mahmoudi, S., Henriksson, S., Corcoran, M., Méndez-Vidal, C., Wiman, K., and Farnebo, M. (2009) Wrap53, a natural p53 antisense transcript required for p53 induction upon DNA damage. *Mol. Cell* **33,** 462–471.
119. Mariner, P., Walters, R., Espinoza, C., Drullinger, L., Wagner, S., Kugel, J., and Goodrich, J. (2008) Human Alu RNA is a modular transacting repressor of mRNA transcription during heat shock. *Mol. Cell* **29,** 499–509.
120. Espinoza, C., Allen, T., Hieb, A., Kugel, J., and Goodrich, J. (2004) B2 RNA binds directly to RNA polymerase II to repress transcript synthesis. *Nat. Struct. Mol. Biol.* **11,** 822–829.
121. Pang, K., Frith, M., and Mattick, J. (2006) Rapid evolution of noncoding RNAs: lack of conservation does not mean lack of function. *Trends Genet.* **22,** 1–5.
122. Taft, R., Glazov, E., Cloonan, N., Simons, C., Stephen, S., Faulkner, G., Lassmann, T., Forrest, A., Grimmond, S., Schroder, K., Irvine, K., Arakawa, T., Nakamura, M., Kubosaki, A., Hayashida, K., Kawazu, C., Murata, M., Nishiyori, H., Fukuda, S., Kawai, J., Daub, C., Hume, D., Suzuki, H., Orlando, V., Carninci, P., Hayashizaki, Y., and Mattick, J. (2009) Tiny RNAs associated with transcription start sites in animals. *Nat. Genet.* **41,** 572–578.
123. Neil, H., Malabat, C., d'Aubenton-Carafa, Y., Xu, Z., Steinmetz, L., and Jacquier, A. (2009) Widespread bidirectional promoters are the major source of cryptic transcripts in yeast. *Nature* **457,** 1038–1042.
124. Cook, P. (2002) Predicting three-dimensional genome structure from transcriptional activity. *Nat. Genet.* **32,** 347–352.
125. Redrup, L., Branco, M., Perdeaux, E., Krueger, C., Lewis, A., Santos, F., Nagano, T., Cobb, B., Fraser, P., and Reik, W. (2009) The long noncoding RNA Kcnq1ot1 organises a lineage-specific nuclear domain for epigenetic gene silencing. *Development* **136,** 525–530.
126. Chaumeil, J., Le Baccon, P., Wutz, A., and Heard, E. (2006) A novel role for Xist RNA in the formation of a repressive nuclear compartment into which genes are recruited when silenced. *Genes Dev.* **20,** 2223–2237.
127. Clemson, C., Hall, L., Byron, M., McNeil, J., and Lawrence, J. (2006) The X chromosome is organized into a gene-rich outer rim and an internal core containing silenced nongenic sequences. *Proc. Natl. Acad. Sci. U. S. A.* **103,** 7688–7693.
128. Marshall, W., Straight, A., Marko, J., Swedlow, J., Dernburg, A., Belmont, A., Murray, A., Agard, D., and Sedat, J. (1997) Interphase chromosomes undergo constrained diffusional motion in living cells. *Curr. Biol.* **7,** 930–939.
